# Poly[(μ_6_-2-methyl-3,5-dinitro­benzoato)potassium]

**DOI:** 10.1107/S1600536810015400

**Published:** 2010-05-08

**Authors:** Muhammad Danish, Iram Saleem, Nazir Ahmad, Wojciech Starosta, Janusz Leciejewicz

**Affiliations:** a Department of Chemistry, University of Sargodha, Sargodha 40100, Pakistan; b Institute of Nuclear Chemistry and Technology, ul. Dorodna 16, 03-195 Warszawa, Poland

## Abstract

In the structure of the title coordination polymer, [K(C_8_H_5_N_2_O_6_)]_*n*_, each ligand bridges six K^+^ cations. The carboxyl­ate group coordinates both bidentately to one K^+^ ion and monodentately to two K^+^ ions, while one nitro group coordinates bidentately to a fourth K^+^ ion. The last two K^+^ ions are coordinated by the remaining nitro group, one in a bidentate fashion, the other monodentately through one O atom. This bridging mode results in a three-dimensional network. The coordination geometry of the K^+^ ion is represented by an irregular KO_9_ polyhedron. Very weak C—H⋯O inter­actions are observed in the crystal structure.

## Related literature

Tin complexes with organic ligands have attracted considerable inter­est due to their biological activity, see, for example: Shahzadi *et al.* (2007 ▶). For the structure of a sodium(I) complex with the 2-methyl-3,5-dinitro-benzoate ligand, see: Danish *et al.* (2010 ▶). 
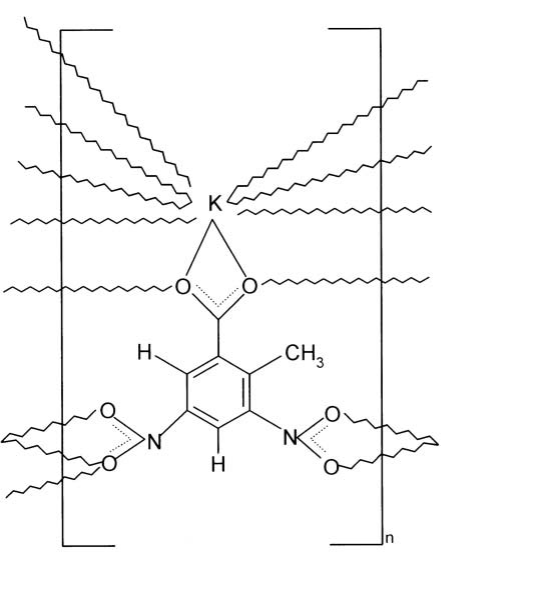


## Experimental

### 

#### Crystal data

[K(C_8_H_5_N_2_O_6_)]
*M* *_r_* = 264.24
Monoclinic, 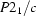

*a* = 8.1632 (16) Å
*b* = 16.998 (3) Å
*c* = 7.0684 (14) Å
β = 90.49 (3)°
*V* = 980.7 (3) Å^3^
*Z* = 4
Mo *K*α radiation
μ = 0.56 mm^−1^
*T* = 293 K
0.43 × 0.32 × 0.22 mm

#### Data collection

Kuma KM-4 four-circle diffractometer
Absorption correction: analytical (*CrysAlis RED*; Oxford Diffraction, 2008 ▶) *T*_min_ = 0.889, *T*_max_ = 0.920
3035 measured reflections
2855 independent reflections
2200 reflections with *I* > 2σ(*I*)
*R*_int_ = 0.033
3 standard reflections every 200 reflections  intensity decay: 0.7%

#### Refinement

*R*[*F* ^2^ > 2σ(*F* ^2^)] = 0.049
*wR*(*F* ^2^) = 0.148
*S* = 1.06
2855 reflections
155 parameters
H-atom parameters constrained
Δρ_max_ = 0.72 e Å^−3^
Δρ_min_ = −0.72 e Å^−3^

### 

Data collection: *KM-4 Software* (Kuma, 1996 ▶); cell refinement: *KM-4 Software*; data reduction: *DATAPROC* (Kuma, 2001 ▶); program(s) used to solve structure: *SHELXS97* (Sheldrick, 2008 ▶); program(s) used to refine structure: *SHELXL97* (Sheldrick, 2008 ▶); molecular graphics: *SHELXTL* (Sheldrick, 2008 ▶); software used to prepare material for publication: *SHELXTL*.

## Supplementary Material

Crystal structure: contains datablocks I, global. DOI: 10.1107/S1600536810015400/ez2207sup1.cif

Structure factors: contains datablocks I. DOI: 10.1107/S1600536810015400/ez2207Isup2.hkl

Additional supplementary materials:  crystallographic information; 3D view; checkCIF report

## Figures and Tables

**Table 1:** Hydrogen-bond geometry (Å, °)

*D*—H⋯*A*	*D*—H	H⋯*A*	*D*⋯*A*	*D*—H⋯*A*
C6—H6⋯O4^i^	0.93	2.59	3.518 (2)	174
C8—H81⋯O4^ii^	0.96	2.84	3.576 (3)	134
C8—H82⋯O2^iii^	0.96	2.78	3.610 (2)	146

Symmetry codes: (i) 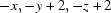; (ii) 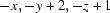; (iii) 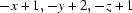.

**Fig. 1.**
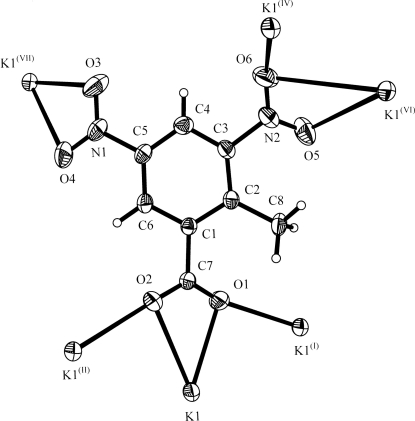
A structural unit of (1) with atom labelling scheme and 50% probability displacement ellipsoids. Symmetry codes: (i) x,-y-3/2,z-1/2; (ii) x,-y+3/2,z+1/2; (iii) -x,y-1/2,-z+3/2; (iv) -x+1,-y+2,-z+1; (v) -x+1,y-1/2,-z+1/2; (vi) -x+1,y+1/2;-z+1/2; (vii) -x,y+1/2,-z+3/2.

**Fig. 2.**
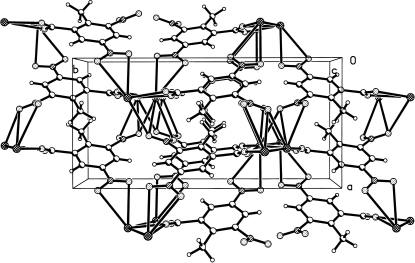
Packing diagram of the structure.

## supplementary crystallographic information

### Comment

Methyl-benzoic acids have been studied as precursors in the synthesis of biologically active tin(IV) complexes (Shahzadi *et al.*, 2007). The structure of compound (1) is a three-dimensional polymeric network in which K^+^ ions are bridged by carboxylate and nitro-group O atoms of the ligand (Fig. 1). The ligand's carboxylate group coordinates bidentately to K1. Its oxygen atoms also coordinate to K1^(i)^ and K1^(ii)^ [symmetry codes: (i) x,-y-3/2,z-1/2; (ii) x,-y+3/2,z+1/2]. The planes formed by atoms K1/O1/K1^(i)^/O2^(i)^ and K1/O2/K1^(ii)^/O1^(ii)^, each with s.u.s of 0.1326 (2) Å, make angles of 8.7 (1)° with the C7/O1/O2 plane forming a zig-zag molecular ribbon. A three-dimensional network (Fig. 2) composed of the ribbons inter-connected by nitro-groups represents the stucture of the title compound. The N1/O3/O4 nitro-group coordinates bidentately to K1^(vii)^; N2/O5/O6 is chelated to the K1^(vi)^, however, the O6 atom is also linked to K1^(iv)^. The carboxylic group C7/O1/O2 makes an angle of 38.0 (1)° to the methylbenzene ring, while the nitro-groups N1/O3/O4 and N2/O5/O6 are oriented at angles of 6.7 (1)° and 35.5 (1)°, respectively. K1 is nine-coordinate with a complicated geometry, while the coordination environment of a Na(I) ion in the complex with the same ligand consists of seven O atoms (Danish *et al.*, 2010). Very weak interactions of the C—H···O type are also operating.

### Experimental

50 ml of aqueous solution containing 0.008 mol of 2-methyl-3,5-dinitro benzoic acid was added dropwise to 50 ml of an aqueous solution of potassium hydroxide (0.008 mol) with constant stirring at room temperature. The mixture was refluxed for 3 hours, then brought to room temperature and concentrated under reduced pressure. A brown solid was purified by repeated crystallization from ethanol-ethyl acetate (1:1) mixture to obtain brown single crystals.

### Refinement

H atoms attached to methyl and benzene-ring C atoms were positioned geometrically (C–H = 0.95–0.98 Å) and refined as riding with *U*_iso_(H) = 1.2–1.5*U*_eq_(C).

### Crystal data

**Table:**

[K(C_8_H_5_N_2_O_6_)]	*F*(000) = 536
*M**_r_* = 264.24	*D*_x_ = 1.790 Mg m^−^^3^
Monoclinic, *P*2_1_/*c*	Mo *K*α radiation, λ = 0.71073 Å
Hall symbol: -P 2ybc	Cell parameters from 25 reflections
*a* = 8.1632 (16) Å	θ = 6–15°
*b* = 16.998 (3) Å	µ = 0.56 mm^−^^1^
*c* = 7.0684 (14) Å	*T* = 293 K
β = 90.49 (3)°	Block, brown
*V* = 980.7 (3) Å^3^	0.43 × 0.32 × 0.22 mm
*Z* = 4	

### Data collection

**Table:**

Kuma KM-4 four-circle diffractometer	2200 reflections with *I* > 2σ(*I*)
Radiation source: fine-focus sealed tube	*R*_int_ = 0.033
graphite	θ_max_ = 30.1°, θ_min_ = 2.4°
profile data from ω/2θ scans	*h* = −11→0
Absorption correction: analytical (*CrysAlis RED*; Oxford Diffraction, 2008)	*k* = 0→23
*T*_min_ = 0.889, *T*_max_ = 0.920	*l* = −9→9
3035 measured reflections	3 standard reflections every 200 reflections
2855 independent reflections	intensity decay: 0.7%

### Refinement

**Table:**

Refinement on *F*^2^	Primary atom site location: structure-invariant direct methods
Least-squares matrix: full	Secondary atom site location: difference Fourier map
*R*[*F*^2^ > 2σ(*F*^2^)] = 0.049	Hydrogen site location: inferred from neighbouring sites
*wR*(*F*^2^) = 0.148	H-atom parameters constrained
*S* = 1.06	*w* = 1/[σ^2^(*F*_o_^2^) + (0.1144*P*)^2^ + 0.0549*P*] where *P* = (*F*_o_^2^ + 2*F*_c_^2^)/3
2855 reflections	(Δ/σ)_max_ = 0.001
155 parameters	Δρ_max_ = 0.72 e Å^−^^3^
0 restraints	Δρ_min_ = −0.72 e Å^−^^3^

### Special details

**Table:**

Geometry. All esds (except the esd in the dihedral angle between two l.s. planes) are estimated using the full covariance matrix. The cell esds are taken into account individually in the estimation of esds in distances, angles and torsion angles; correlations between esds in cell parameters are only used when they are defined by crystal symmetry. An approximate (isotropic) treatment of cell esds is used for estimating esds involving l.s. planes.
Refinement. Refinement of *F*^2^ against ALL reflections. The weighted *R*-factor wR and goodness of fit *S* are based on *F*^2^, conventional *R*-factors *R* are based on *F*, with *F* set to zero for negative *F*^2^. The threshold expression of *F*^2^ > σ(*F*^2^) is used only for calculating *R*-factors(gt) etc. and is not relevant to the choice of reflections for refinement. *R*-factors based on *F*^2^ are statistically about twice as large as those based on *F*, and *R*- factors based on ALL data will be even larger.

### Fractional atomic coordinates and isotropic or equivalent isotropic displacement parameters (Å^2^)

**Table:**

	*x*	*y*	*z*	*U*_iso_*/*U*_eq_	
K1	0.30646 (5)	0.70455 (2)	0.53719 (5)	0.03281 (15)	
C1	0.24609 (16)	0.97819 (8)	0.52276 (19)	0.0220 (3)	
C7	0.26751 (18)	0.88921 (9)	0.5373 (2)	0.0246 (3)	
C2	0.30932 (17)	1.02265 (9)	0.3727 (2)	0.0233 (3)	
C6	0.16004 (17)	1.01430 (9)	0.6686 (2)	0.0255 (3)	
H6	0.1179	0.9844	0.7670	0.031*	
C3	0.28318 (19)	1.10416 (9)	0.3816 (2)	0.0266 (3)	
O1	0.25937 (19)	0.85008 (8)	0.38860 (17)	0.0395 (3)	
O6	0.39480 (19)	1.22199 (8)	0.2829 (2)	0.0471 (4)	
O2	0.28968 (18)	0.86252 (8)	0.69897 (17)	0.0373 (3)	
N1	0.04619 (18)	1.13215 (9)	0.8188 (2)	0.0346 (3)	
N2	0.34771 (18)	1.15683 (9)	0.2343 (2)	0.0334 (3)	
C5	0.13803 (18)	1.09462 (9)	0.6657 (2)	0.0270 (3)	
O5	0.3523 (2)	1.13326 (10)	0.0711 (2)	0.0526 (4)	
C8	0.4096 (2)	0.98522 (10)	0.2191 (2)	0.0319 (3)	
H81	0.3405	0.9739	0.1120	0.048*	
H83	0.4573	0.9373	0.2656	0.048*	
H82	0.4951	1.0207	0.1819	0.048*	
C4	0.20004 (19)	1.14170 (10)	0.5248 (2)	0.0295 (3)	
H4	0.1866	1.1960	0.5261	0.035*	
O3	0.0391 (2)	1.20350 (9)	0.8230 (3)	0.0503 (4)	
O4	−0.0210 (2)	1.08967 (10)	0.9335 (2)	0.0533 (4)	

### Atomic displacement parameters (Å^2^)

**Table:**

	*U*^11^	*U*^22^	*U*^33^	*U*^12^	*U*^13^	*U*^23^
K1	0.0473 (3)	0.0273 (2)	0.0239 (2)	−0.00228 (13)	0.00711 (15)	0.00005 (11)
C1	0.0202 (6)	0.0239 (6)	0.0219 (6)	0.0009 (5)	0.0005 (5)	−0.0003 (5)
C7	0.0229 (6)	0.0250 (7)	0.0259 (7)	0.0011 (5)	0.0041 (5)	0.0009 (5)
C2	0.0202 (6)	0.0269 (7)	0.0228 (6)	−0.0014 (5)	0.0016 (5)	−0.0004 (5)
C6	0.0217 (6)	0.0314 (8)	0.0234 (6)	0.0004 (5)	0.0042 (5)	0.0004 (5)
C3	0.0250 (7)	0.0270 (7)	0.0280 (7)	−0.0014 (5)	0.0026 (5)	0.0045 (5)
O1	0.0608 (9)	0.0291 (6)	0.0285 (6)	0.0017 (6)	0.0010 (5)	−0.0041 (5)
O6	0.0453 (8)	0.0310 (7)	0.0650 (10)	−0.0058 (5)	0.0100 (7)	0.0082 (6)
O2	0.0520 (8)	0.0329 (6)	0.0270 (6)	0.0053 (5)	0.0035 (5)	0.0063 (5)
N1	0.0266 (7)	0.0394 (8)	0.0381 (7)	0.0041 (5)	0.0069 (5)	−0.0106 (6)
N2	0.0282 (6)	0.0336 (7)	0.0385 (7)	−0.0012 (5)	0.0023 (6)	0.0121 (6)
C5	0.0212 (6)	0.0312 (7)	0.0288 (7)	0.0029 (5)	0.0038 (5)	−0.0050 (6)
O5	0.0650 (10)	0.0608 (10)	0.0321 (7)	−0.0112 (8)	0.0014 (7)	0.0126 (6)
C8	0.0306 (7)	0.0386 (9)	0.0268 (7)	−0.0033 (6)	0.0107 (6)	−0.0039 (6)
C4	0.0261 (7)	0.0262 (7)	0.0361 (8)	0.0022 (5)	0.0028 (6)	−0.0002 (6)
O3	0.0412 (8)	0.0410 (8)	0.0688 (10)	−0.0005 (5)	0.0146 (7)	−0.0228 (7)
O4	0.0585 (10)	0.0587 (9)	0.0432 (8)	0.0134 (7)	0.0271 (7)	0.0030 (7)

### Geometric parameters (Å, °)

**Table:**

K1—O2^i^	2.6511 (13)	C3—C4	1.380 (2)
K1—O1^ii^	2.6826 (13)	C3—N2	1.473 (2)
K1—O1	2.7133 (14)	O1—K1^i^	2.6826 (13)
K1—O2	2.9221 (14)	O6—N2	1.221 (2)
K1—O3^iii^	2.9974 (18)	O6—K1^iv^	3.0116 (18)
K1—O6^iv^	3.0115 (18)	O6—K1^vi^	3.3541 (19)
K1—O4^iii^	3.0485 (18)	O2—K1^ii^	2.6511 (13)
K1—O5^v^	3.1388 (19)	N1—O3	1.214 (2)
K1—C7	3.1548 (17)	N1—O4	1.220 (2)
K1—N1^iii^	3.2998 (16)	N1—C5	1.468 (2)
K1—O6^v^	3.3541 (19)	N1—K1^vii^	3.2997 (16)
K1—K1^i^	3.8572 (7)	N2—O5	1.222 (2)
C1—C6	1.395 (2)	C5—C4	1.377 (2)
C1—C2	1.4042 (19)	O5—K1^vi^	3.1388 (19)
C1—C7	1.526 (2)	C8—H81	0.9600
C7—O2	1.2414 (19)	C8—H83	0.9600
C7—O1	1.2450 (19)	C8—H82	0.9600
C2—C3	1.403 (2)	C4—H4	0.9300
C2—C8	1.506 (2)	O3—K1^vii^	2.9973 (18)
C6—C5	1.377 (2)	O4—K1^vii^	3.0485 (17)
C6—H6	0.9300		
			
O2^i^—K1—O1^ii^	132.80 (4)	O3^iii^—K1—K1^i^	108.17 (4)
O2^i^—K1—O1	92.12 (4)	O6^iv^—K1—K1^i^	102.38 (4)
O1^ii^—K1—O1	130.77 (4)	O4^iii^—K1—K1^i^	108.96 (4)
O2^i^—K1—O2	138.23 (4)	O5^v^—K1—K1^i^	85.65 (4)
O1^ii^—K1—O2	87.03 (4)	C7—K1—K1^i^	66.58 (3)
O1—K1—O2	46.12 (4)	N1^iii^—K1—K1^i^	116.03 (4)
O2^i^—K1—O3^iii^	104.67 (6)	O6^v^—K1—K1^i^	48.76 (3)
O1^ii^—K1—O3^iii^	63.25 (5)	C6—C1—C2	120.82 (14)
O1—K1—O3^iii^	90.14 (4)	C6—C1—C7	116.39 (13)
O2—K1—O3^iii^	80.16 (5)	C2—C1—C7	122.79 (12)
O2^i^—K1—O6^iv^	126.47 (5)	O2—C7—O1	126.02 (16)
O1^ii^—K1—O6^iv^	82.79 (5)	O2—C7—C1	116.09 (13)
O1—K1—O6^iv^	84.09 (5)	O1—C7—C1	117.88 (14)
O2—K1—O6^iv^	59.62 (4)	O2—C7—K1	67.84 (9)
O3^iii^—K1—O6^iv^	128.64 (5)	O1—C7—K1	58.19 (9)
O2^i^—K1—O4^iii^	75.65 (5)	C1—C7—K1	176.06 (10)
O1^ii^—K1—O4^iii^	66.40 (5)	C3—C2—C1	116.14 (13)
O1—K1—O4^iii^	120.29 (5)	C3—C2—C8	122.20 (13)
O2—K1—O4^iii^	121.57 (5)	C1—C2—C8	121.51 (14)
O3^iii^—K1—O4^iii^	41.55 (5)	C5—C6—C1	119.43 (14)
O6^iv^—K1—O4^iii^	148.60 (5)	C5—C6—H6	120.3
O2^i^—K1—O5^v^	69.70 (5)	C1—C6—H6	120.3
O1^ii^—K1—O5^v^	103.11 (5)	C4—C3—C2	124.41 (14)
O1—K1—O5^v^	112.37 (5)	C4—C3—N2	114.73 (15)
O2—K1—O5^v^	119.66 (5)	C2—C3—N2	120.86 (14)
O3^iii^—K1—O5^v^	156.64 (5)	C7—O1—K1^i^	163.63 (12)
O6^iv^—K1—O5^v^	62.99 (5)	C7—O1—K1	98.86 (10)
O4^iii^—K1—O5^v^	116.73 (5)	K1^i^—O1—K1	91.26 (4)
O2^i^—K1—C7	115.06 (4)	N2—O6—K1^iv^	138.32 (12)
O1^ii^—K1—C7	109.20 (4)	N2—O6—K1^vi^	87.65 (10)
O1—K1—C7	22.95 (4)	K1^iv^—O6—K1^vi^	74.37 (4)
O2—K1—C7	23.17 (4)	C7—O2—K1^ii^	173.37 (12)
O3^iii^—K1—C7	84.88 (4)	C7—O2—K1	89.00 (10)
O6^iv^—K1—C7	70.65 (4)	K1^ii^—O2—K1	87.45 (4)
O4^iii^—K1—C7	124.07 (5)	O3—N1—O4	123.59 (16)
O5^v^—K1—C7	118.28 (5)	O3—N1—C5	118.47 (16)
O2^i^—K1—N1^iii^	94.55 (5)	O4—N1—C5	117.93 (16)
O1^ii^—K1—N1^iii^	56.83 (5)	O3—N1—K1^vii^	65.06 (9)
O1—K1—N1^iii^	109.77 (5)	O4—N1—K1^vii^	67.48 (10)
O2—K1—N1^iii^	100.25 (4)	C5—N1—K1^vii^	147.35 (11)
O3^iii^—K1—N1^iii^	21.56 (4)	O6—N2—O5	123.42 (15)
O6^iv^—K1—N1^iii^	136.87 (4)	O6—N2—C3	117.79 (15)
O4^iii^—K1—N1^iii^	21.69 (4)	O5—N2—C3	118.79 (15)
O5^v^—K1—N1^iii^	135.22 (5)	C6—C5—C4	122.57 (14)
C7—K1—N1^iii^	106.43 (4)	C6—C5—N1	119.15 (14)
O2^i^—K1—O6^v^	57.45 (4)	C4—C5—N1	118.28 (15)
O1^ii^—K1—O6^v^	140.00 (5)	N2—O5—K1^vi^	97.94 (12)
O1—K1—O6^v^	76.10 (4)	C2—C8—H81	109.5
O2—K1—O6^v^	102.69 (4)	C2—C8—H83	109.5
O3^iii^—K1—O6^v^	156.18 (5)	H81—C8—H83	109.5
O6^iv^—K1—O6^v^	69.96 (4)	C2—C8—H82	109.5
O4^iii^—K1—O6^v^	131.53 (4)	H81—C8—H82	109.5
O5^v^—K1—O6^v^	38.52 (4)	H83—C8—H82	109.5
C7—K1—O6^v^	89.20 (4)	C5—C4—C3	116.61 (15)
N1^iii^—K1—O6^v^	151.93 (4)	C5—C4—H4	121.7
O2^i^—K1—K1^i^	49.19 (3)	C3—C4—H4	121.7
O1^ii^—K1—K1^i^	171.19 (3)	N1—O3—K1^vii^	93.38 (10)
O1—K1—K1^i^	44.05 (3)	N1—O4—K1^vii^	90.82 (11)
O2—K1—K1^i^	89.48 (3)		

Symmetry codes: (i) *x*, −*y*+3/2, *z*−1/2; (ii) *x*, −*y*+3/2, *z*+1/2; (iii) −*x*, *y*−1/2, −*z*+3/2; (iv) −*x*+1, −*y*+2, −*z*+1; (v) −*x*+1, *y*−1/2, −*z*+1/2; (vi) −*x*+1, *y*+1/2, −*z*+1/2; (vii) −*x*, *y*+1/2, −*z*+3/2.

### Hydrogen-bond geometry (Å, °)

**Table:**

*D*—H···*A*	*D*—H	H···*A*	*D*···*A*	*D*—H···*A*
C6—H6···O4^viii^	0.93	2.59	3.518 (2)	174
C8—H81···O4^ix^	0.96	2.84	3.576 (3)	134
C8—H82···O2^iv^	0.96	2.78	3.610 (2)	146

Symmetry codes: (viii) −*x*, −*y*+2, −*z*+2; (ix) −*x*, −*y*+2, −*z*+1; (iv) −*x*+1, −*y*+2, −*z*+1.

## References

[bb1] Danish, M., Saleem, I., Ahmad, N., Raza, A. R., Starosta, W. & Leciejewicz, J. (2010). *Acta Cryst.* E**66**, m137. 10.1107/S1600536810000498 PMC2979836 21579619

[bb2] Kuma (1996). *KM-4 Software* Kuma Diffraction Ltd, Wrocław, Poland.

[bb3] Kuma (2001). *DATAPROC* Kuma Diffraction Ltd, Wrocław, Poland.

[bb4] Oxford Diffraction (2008). *CrysAlis RED* Oxford Diffraction Ltd, Yarnton, England

[bb5] Shahzadi, S., Shahid, K. & Ali, S. (2007). *Russ. J. Coord. Chem.* **33**, 403–411.

[bb6] Sheldrick, G. M. (2008). *Acta Cryst.* A**64**, 112–122. 10.1107/S0108767307043930 18156677

